# Supplementary material for: Relationship between self-efficacy, adversity quotient, COVID-19-related stress and academic performance among the undergraduate students: A protocol for a systematic review
Source: PLoS One. 2022 Dec 1;17(12):e0278635. doi: 10.1371/journal.pone.0278635 (PMC9714802; doi:10.1371/journal.pone.0278635)
Supplement: Attachment — Submitted filename: Response to Reviewers.docx [file pone.0278635.s003.docx]

**Rebuttal Letter**

___________________________________________________________________________

Thank you to the editor and the reviewers for their comments. May the responses address the comments accordingly and the manuscript would be potentially considered for publication. The responses to all comments are highlighted in the following section.

Sincerely,

On behalf of all authors,

Noh Amit

**Reviewer #3**

1. **Have the authors described where all data underlying the findings will be made available when the study is complete? NO.**

The description on the availability of data was inserted on lines 320-321.

**Revision:** The data supporting the findings of this study will be made available within the article and/or its supplementary materials.

2. **In the abstract at lines 27-29, the search terms used should be in apostrophes, as they are in the text - lines 225-227.**

The apostrophes for search terms were inserted on lines 27-28.

**Revision:** …‘self-efficacy’ AND ‘adversity quotient’ AND ‘stress’ AND ‘academic performance’ AND ‘student’ AND ‘COVID-19 pandemic’.

3. **In lines 67-69 the same information are repeated in two consecutive sentences.**

The repeated sentence (i.e., The stress level was measured by using Student-life Stress Inventory) was removed from the paragraph on lines 65-69.

**Revision:** Furthermore, similar study was conducted by Putri and Ariana in Indonesian context where the stress level was measured by Student-life Stress Inventory [8]. The findings show that out of 252 first-year undergraduate students studied, 110 of them (43.6%) reported low stress, 89 respondents reported moderate stress (35.3%), and 53 respondents (21%) reported high stress.

4. **In lines 84-111, the figures in brackets will be better understood if the authors choose to present the percentages themselves, or replace the comma with a dash - e.g. (173 - 89%).**

The figures and percentages in brackets were revised as follows.

**80-88:**	There are various changes in terms of educational approaches which potentially lead to difficulty in concentrating on academic work. Son et al. [9] found that most of the students (173 - 89%) stated that their home is distractive. For them, home is more appropriate place to than to study (79 - 46%). In fact, students were easier to be bothered by their family members at home. Other factors influencing students’ concentration were social media, internet, and video games (19 - 11%). Some students (18 - 10%) mentioned that online classes were subject to diversion due to social interaction deficiency and continuous attention to computer screen. In addition, monotone life patterns mentioned by some students could negatively affect concentration on academic work (5 - 3%).

**95 – 99:**	Son et al. [9] stated that most students (167 - 86%) answered that the COVID-19 pandemic increased the social isolation. Over half of students (91 - 54%) stated that it limited their interactions with friends significantly. Some students (52 - 31%) stated that they worried about a lack of in-person interactions. Others (9 - 5%) mentioned that disturbance to their outdoor activities have affected their mental health.

**100-110:**	Furthermore, in 2020, Son et al [9] found that majority of students (159 - 82%) concerned on their academic performance impacted by the COVID-19 pandemic. Fear of lower performance and delay in completion of studies are also the reasons to induce stress among students during COVID-19. The biggest challenge was the transition to classes through online platforms (61 - 38%). In particular, students concerned on sudden changes in the syllabus, the quality of the classes, technical issues with online applications, and the difficulty of online learning. Some students (36 - 23%) worried about progress in research and class projects because of social restrictions to keep social distancing and the lack of physical interactions with other students. Others (23 - 14%) stated the uncertainty about their grades on the learning through online platforms to be a major stressor. In addition, others (12 - 8%) indicated their lower motivation to learn and tendency to procrastinate.

5. **In line 314, the sentence should start with a capital letter.**

The capital letter was inserted in the revision of this sentence (on lines 313-314).

**Revision:** The articles which will be selected for this study are the articles published between December 2019 until August 2022.

**Additional Review:**

1. **Please upload a copy of Figure 1 which you refer to in your text on page 11. Or if the figure is no longer to be included as part of the submission please remove all reference to it within the text.**

As the guidelines say, which:

- each figure caption should appear directly after the paragraph in which they were first cited, and
- all figures should be uploaded separately as individual files,

**Revision:**

- the caption has added on page 11 lines 241 as “**Fig 1. PRISMA Flow Chart.”**,
- the figure has uploaded and attached as individual files namely **“Fig1.tiff”**.
